# Prognostic Role of Multiparametric Cardiac Magnetic Resonance in Neo Transfusion-Dependent Thalassemia

**DOI:** 10.3390/jcm13051281

**Published:** 2024-02-23

**Authors:** Antonella Meloni, Laura Pistoia, Paolo Ricchi, Aurelio Maggio, Valerio Cecinati, Filomena Longo, Francesco Sorrentino, Zelia Borsellino, Alessandra Salvo, Vincenza Rossi, Emanuele Grassedonio, Gennaro Restaino, Stefania Renne, Riccardo Righi, Vincenzo Positano, Filippo Cademartiri

**Affiliations:** 1 Bioengineering Unit, Fondazione G. Monasterio CNR-Regione Toscana, 56124 Pisa, Italy; antonella.meloni@ftgm.it (A.M.); positano@ftgm.it (V.P.); 2 Department of Radiology, Fondazione G. Monasterio CNR-Regione Toscana, 56124 Pisa, Italy; laura.pistoia@ftgm.it; 3 Unità Operativa Complessa Ricerca Clinica, Fondazione G. Monasterio CNR-Regione Toscana, 56124 Pisa, Italy; 4 Unità Operativa Semplice Dipartimentale Malattie Rare del Globulo Rosso, Azienda Ospedaliera di Rilievo Nazionale “A. Cardarelli”, 80131 Napoli, Italy; paolo.ricchi@aocardarelli.it; 5 Ematologia II con Talassemia, Ospedale “V. Cervello”, 90100 Palermo, Italy; aurelio.maggio@ospedaliriunitipalermo.it; 6 Struttura Semplice di Microcitemia, Ospedale “SS. Annunziata”, 74123 Taranto, Italy; valerio.cecinati@als.taranto.it; 7 Unità Operativa Day Hospital della Talassemia e delle Emoglobinopatie, Azienda Ospedaliero-Universitaria “S. Anna”, 44124 Cona, FE, Italy; filomena.longo@ospfe.it; 8 Unità Operativa Semplice Dipartimentale Day Hospital Talassemici, Ospedale “Sant’Eugenio”, 00143 Rome, Italy; sorrentino.francesco@aslrmc.it; 9 Unità Operativa Complessa Ematologia con Talassemia, ARNAS Civico “Benfratelli-Di Cristina”, 90134 Palermo, Italy; zelia.borsellino@arnascivico.it; 10 Operativa Semplice Talassemia, Presidio Ospedaliero “Umberto I”, 96100 Siracusa, Italy; gaetasalvo@gmail.com; 11 Unità Operativa Complessa Ematologia, Ospedale di Cosenza, 87100 Cosenza, Italy; enza.rossi@libero.it; 12 Sezione di Scienze Radiologiche, Dipartimento di Biopatologia e Biotecnologie Mediche, Policlinico “Paolo Giaccone”, 90127 Palermo, Italy; egrassedonio@gmail.com; 13 Unità Operativa Complessa Radiodiagnostica, Gemelli Molise SpA, Fondazione di Ricerca e Cura “Giovanni Paolo II”, 86100 Campobasso, Italy; gennaro.restaino@gemellimolise.it; 14 Struttura Complessa di Cardioradiologia-UTIC, Presidio Ospedaliero “Giovanni Paolo II”, 88046 Lamezia Terme, CZ, Italy; stefania.renne@virgilio.it; 15 Diagnostica per Immagini e Radiologia Interventistica, Ospedale del Delta, 44023 Lagosanto, FE, Italy; riccardo.righi@ausl.fe.it

**Keywords:** thalassemia, cardiovascular magnetic resonance, cardiovascular complications, prognosis

## Abstract

Background: We prospectively evaluated the predictive value of multiparametric cardiac magnetic resonance (CMR) for cardiovascular complications in non-transfusion-dependent β-thalassemia (β-NTDT) patients who started regular transfusions in late childhood/adulthood (neo β-TDT). Methods: We considered 180 patients (38.25 ± 11.24 years; 106 females). CMR was used to quantify cardiac iron overload, biventricular function, and atrial dimensions, and to detect left ventricular (LV) replacement fibrosis. Results: During a mean follow-up of 76.87 ± 41.60 months, 18 (10.0%) cardiovascular events were recorded: 2 heart failures, 13 arrhythmias (10 supraventricular), and 3 cases of pulmonary hypertension. Right ventricular (RV) end-diastolic volume index (EDVI), RV mass index (MI), LV replacement fibrosis, and right atrial (RA) area index emerged as significant univariate prognosticators of cardiovascular complications. The low number of events prevented us from performing a multivariable analysis including all univariable predictors simultaneously. Firstly, a multivariable analysis including the two RV size parameters (mass and volume) was carried out, and only the RV MI was proven to independently predict cardiovascular diseases. Then, a multivariable analysis, including RV MI, RA atrial area, and LV replacement fibrosis, was conducted. In this model, RV MI and LV replacement fibrosis emerged as independent predictors of cardiovascular outcomes (RV MI: hazard ratio (HR) = 1.18; LV replacement fibrosis: HR = 6.26). Conclusions: Our results highlight the importance of CMR in cardiovascular risk stratification.

## 1. Introduction

Thalassemias are inherited hemoglobinopathies that result from defective synthesis of the beta (β-thalassemia) or alpha (α-thalassemia) globin chains of adult hemoglobin [1,2]. The traditional classification system for thalassemia categorizes patients into three different phenotypes based on the severity of the condition and the specific genetic mutations involved [3]. Thalassemia major (TM) is the most severe form of the disease, characterized by early presentation with severe anemia. In contrast, thalassemia minor is the mildest form of the disease, and affected patients are usually asymptomatic or have mild anemia. Thalassemia intermedia (TI) represents an intermediate form of the disease in terms of severity.

An alternative classification system for clinically relevant forms of thalassemia (excluding carriers and minor/trait patients) was introduced approximately ten years ago [4]. It was based on transfusion requirements because it was recognized that the receipt of transfusions had significant implications for the underlying pathophysiology and management needs. The term transfusion-dependent thalassemia (TDT) is primarily used for patients with β-TM who are highly dependent on transfusions for survival [5]. Non-transfusion-dependent thalassemia (NTDT) refers to patients who do not require lifelong and regular blood transfusions to survive. NTDT encompasses several clinically diverse forms, including β-thalassemia intermedia (β-TI), hemoglobin E/β-thalassemia, and α-thalassemia intermedia (hemoglobin H disease) [4,6]. Some NTDT patients may require regular transfusion therapy to manage their condition effectively [7]. Indeed, transfusion therapy has been shown to protect against various complications, including thrombosis, extramedullary hematopoiesis, pulmonary hypertension (PH), cholelithiasis, and leg ulcers, and it may also help to reduce intestinal iron absorption [8,9,10]. It has been suggested that these patients should be referred to as neo-TDT [11] to distinguish them from those who have experienced TDT since diagnosis, with long-term and lifelong exposure to transfusional iron overload and iron chelation therapy. The human body lacks an efficient natural physiological mechanism for eliminating excess iron [12], and excess iron resulting from multiple blood transfusions can accumulate in several organs throughout the body, potentially causing organ damage and dysfunction [13,14,15].

The pathophysiological characteristics of the heart have been extensively studied in β-TDT, but not in neo β-TDT. Due to its multiparametric nature, cardiac magnetic resonance (CMR) is a powerful and comprehensive tool for assessing both structural and functional cardiac abnormalities. Thanks to its excellent accuracy and repeatability, CMR is currently the gold standard for the quantitative assessment of systolic function and cardiac chamber size [16,17]. Moreover, CMR possesses a unique capability for in vivo myocardial tissue characterization; it can detect alterations in tissue composition, like edema or fibrosis, and it can differentiate and quantify various substances present in the myocardium, including iron [18,19]. In particular, the T2* technique is the gold standard for the non-invasive and reproducible quantification of myocardial iron overload [20], validated against histopathological features [21,22].

A recent multicenter CMR study demonstrated that, compared to β-NTDT patients, neo β-TDT patients had more cardiac iron burden (lower T2* values) but less dilated ventricles [23], suggesting that blood transfusions can partially mitigate the adverse hemodynamic consequences of anemia. The hearts of thalassemic patients respond to chronic anemia by increasing the stroke volume and reducing the peripheral vascular resistance [24]. To accommodate the resulting increased workload, the cardiac chambers, especially the ventricles, may dilate or enlarge [25,26]. These are often referred to as volume-loaded ventricles. These compensatory mechanisms can put extra strain on the heart over time, and, if not managed appropriately they may lead to complications.

There is currently a lack of prospective data in the existing literature about the role of multiparametric CMR findings, including assessments of heart iron levels, cardiac function, and replacement myocardial fibrosis, in predicting cardiovascular outcomes in patients with neo β-TDT.

Consequently, the objective of this multicenter study was to prospectively investigate the predictive value of CMR parameters for cardiovascular complications in neo β-TDT patients.

## 2. Materials and Methods

### 2.1. Patient Population

The Myocardial Iron Overload in Thalassemia (MIOT) Network was a collaborative project among more than 60 hematological centers and 10 magnetic resonance imaging (MRI) centers performing MRI examinations according to uniform, standardized, and validated procedures [27]. The inclusion criteria of the MIOT Network were as follows: (1) male and female patients of all ages, with thalassemia or sickle-cell disease, requiring the quantification of cardiac and hepatic iron levels by MRI; (2) written informed consent; (3) signed consent for the use and sharing of protected health information; (4) no absolute contraindications to MRI.

All centers were linked by a shared database, configured to collect the clinical–anamnestic history of the patients from birth to the date of the first MRI scan. According to the protocol, patients repeated the MRI every 18 ± 3 months. The clinical, instrumental, and laboratory data in the database were updated in correspondence with each MRI scan.

Among all patients with hemoglobinopathies consecutively enrolled in the MIOT Network, we selected those with β-NTDT who had started regular transfusions (every 3 weeks to 3 months) before the baseline MRI, performed between April 2006 and May 2014. The mean age of the 196 neo β-TDT patients was 38.76 ± 11.33 years, and 111 (56.6%) were females.

This study complied with the Declaration of Helsinki and was approved by the institutional ethics committee. Written informed consent was provided by every patient.

### 2.2. Magnetic Resonance Imaging

MRI exams were performed using 1.5 T scanners from three main vendors (GE Healthcare, Milwaukee, WI, USA; Philips, Best, the Netherlands; Siemens Healthineers, Erlangen, Germany), equipped with phased-array coils. End-expiration breath-holding and ECG gating were used.

The T2* technique was employed for iron overload assessment. A single mid-hepatic slice [28] and three parallel short-axis views (basal, medium, and apical) of the left ventricle (LV) [29] were acquired with gradient-echo multi-echo sequences. Analysis of T2* images was carried out with the use of a custom-written, previously validated software platform (HIPPOMIOT^®^ Version 1.0, Consiglio Nazionale delle Ricerche and Fondazione Toscana Gabriele Monasterio, Pisa, Italy) [30]. The hepatic T2* value was measured in a large region of interest (ROI) of standard dimensions, defined in a homogeneous section of parenchyma without blood vessels and distant from those areas more prone to susceptibility artifacts [28]. The calibration curve introduced by Wood et al. [31] was employed to convert liver T2* values into liver iron concentration (LIC). The software provided the T2* values for all 16 segments of the LV, according to the standard American Heart Association (AHA)/American College of Cardiology (ACC) model [32]. An appropriate correction map was applied to compensate for susceptibility and geometric artifacts [30]. The global heart T2* value was obtained as the average of all segmental values.

For the quantification of cardiac size and function, cine images were acquired using a breath-holding, balanced, steady-state free precession (SSFP) sequence in long-axis views (2, 4, and 3 chambers), as well as in contiguous short-axis slices (slice thickness = 8 mm), covering the entire LV and right ventricle (RV) from the annulus of the atrioventricular valves to the apex, with 30 phases per cardiac cycle. The short-axis stack was analyzed in a standard manner [33,34] by experienced observers. Epicardial and endocardial borders were semi-automatically defined in the end-diastolic and end-systolic phases in all relevant slices. The interventricular septum was considered to be part of the LV. Papillary muscles and trabeculations of the LV and RV cavities were considered to be part of the LV and RV cavity volumes. End-diastolic volume (EDV) and end-systolic volume (ESV) were determined without geometric assumptions using Simpson’s rule. The ejection fraction (EF) was given by the ratio between the stroke volume (difference between EDV and ESV) and the EDV. The ventricular wall mass was obtained by multiplying the volume of the myocardium by its specific weight (1.05 g/cm^3^). The left and right atrial areas were measured from the four-chamber view projection in the ventricular end-systolic phase. Biventricular volumes and masses and biatrial areas were indexed to the body surface area (BSA).

To detect the presence of LV replacement fibrosis, late gadolinium enhancement (LGE) images at matching cine-image slice locations and slice thicknesses (8 mm) were acquired 10 to 18 min after intravenous administration of gadobutrol (Gadovist^®^; Bayer Schering Pharma; Berlin, Germany) at the standard dose of 0.2 mmol/kg, using a fast gradient-echo inversion recovery T1-weighted sequence. In thalassemia patients, the LGE technique has been proven to be safe [35]. LGE imaging was not performed in patients with a glomerular filtration rate < 30 mL/min/1.73 m^2^ or in patients who refused the contrast medium administration. LGE was considered to be present if observed in two different imaging planes [36]. The extent of the LGE areas was quantified using a previously validated software platform [36].

### 2.3. Diagnostic Criteria and Follow-Up

A T2* measurement of 20 ms was taken as a “conservative” normal value for segmental and global heart T2* values [21,30,37].

An MRI LIC ≥ 3 mg/g/dw was indicative of significant iron load [38].

The follow-up date coincided with the date of the last available MRI. In cases where follow-up MRIs were not performed, the caring hematologist filled out a case report form, which outlined the patient’s outcomes from the baseline MRI until September 2018.

The outcome of this study was the incidence of cardiovascular complications, defined as a composite of heart failure (HF), arrhythmias, and pulmonary hypertension (PH). HF was identified based on symptoms, signs, biomarkers, and instrumental parameters, according to the current guidelines [39]. Arrhythmias were diagnosed if documented by electrocardiogram (ECG) or 24-hour Holter ECG, and if requiring specific medications. Arrhythmias were classified in accordance with the AHA/ACC guidelines. The diagnosis of PH was made when the trans-tricuspid velocity jet on transthoracic echocardiogram exceeded 3.2 m/s [40], in the presence of relevant signs and symptoms. If a patient developed more than one complication, only the first one was taken into account.

### 2.4. Statistical Analysis

All data were analyzed using the SPSS version 27.0 (IBM Corp., Armonk, NY, USA) and MedCalc version 19.8 (MedCalc Software Ltd., Ostend, Belgium) statistical packages.

Continuous variables were represented as the mean ± standard deviation (SD). Categorical variables were expressed as frequencies and percentages.

The Kolmogorov–Smirnov test was employed to evaluate the normality of the distribution of the continuous variables.

The comparisons between two groups were performed using the independent-samples *t*-test for continuous variables with normal distribution, the Mann–Whitney U test for continuous variables that did not adhere to a normal distribution, and the χ^2^ test for categorical variables.

Correlation analysis was performed using Pearson’s or Spearman’s tests, where appropriate.

The Cox proportional hazards model was employed to investigate the potential link between the prognostic variables of interest and the study’s outcome. Each prognostic variable was tested individually, and only those variables showing statistical significance in the univariable analysis were placed in the multivariate model. Any variables that did not contribute significantly to improving the model’s performance were removed from the final analysis. The results were presented as hazard ratios (HRs) with 95% confidence intervals (CIs).

Kaplan–Meier curves were generated to examine the development of an outcome over time in relation to each significant prognostic variable. The log-rank test was employed to compare the differences between various groups or strata in the Kaplan–Meier analyses.

Receiver operating characteristic (ROC) curve analysis was used to find the cutoff value that maximized the balance between sensitivity and specificity for the clinical variables related to the study’s endpoint.

In all tests, a 2-tailed probability value of 0.05 was considered statistically significant.

## 3. Results

### 3.1. Baseline Data

Sixteen patients were excluded from this study because a cardiac complication (three HFs, six arrhythmias, four PH cases, two HFs + arrhythmias, and one HF + PH) was present at the baseline MRI. These patients were significantly older than patients without an active cardiovascular complication (44.58 ± 11.04 years vs. 38.25 ± 11.24 years; *p* = 0.032).

Table 1 summarizes the baseline demographic, clinical, and MRI features of the 180 considered neo-TDT patients. The patients started regular transfusions at a mean age of 17.64 ± 16.61 years, and the mean frequency of transfusions, intended as the number of transfusional units in the 12 months before the MRI scan, was 26.76 ± 10.19. A total of 94.4% of patients were chelated. Among the 170 chelated patients, 62 received deferoxamine as monotherapy, 45 received deferiprone as monotherapy, 40 received deferasirox as monotherapy, 18 received combined deferoxamine + deferiprone therapy, and 5 received sequential deferoxamine/deferiprone therapy.

Hepatic and myocardial iron overload were detected in 73.3% and 11.7% of patients, respectively.

The contrast medium was administered in 125 (69.4%) patients, of whom 26 (20.8%) had replacement myocardial fibrosis. Twenty-four (92.3%) had a non-ischemic pattern of LGE, while an ischemic pattern was found only in two patients. The average number of segments with LGE per patient was 2.81 ± 1.60. LGE affected the septal region in 80.8% of the patients.

### 3.2. Characterization of Patients Who Experienced a Cardiovascular Event

The mean follow-up time was 76.87 ± 41.60 months (median = 75.12 months). Cardiovascular events were recorded in 18 (10.0%) patients: 2 HFs, 13 arrhythmias, and 3 cases of PH. The incidence of globally considered cardiac complications was 1.56%/year (95% CI = 0.93%/year to 2.47%/year), while the incidence of HF, arrhythmias, and PH was 0.17%/year (95% CI = 0.02%/year to 0.63%/year), 1.13%/year (95% CI = 0.60%/year to 1.93%/year), and 0.26%/year (95% CI = 0.05%/year to 0.76%/year), respectively. The mean time from the first MRI to the development of a cardiac complication was 39.82 ± 32.46 months.

Among the arrhythmias, the supraventricular arrhythmias (atrial fibrillation and atrial flutter) were the most common type (10/13 = 76.9%). The incidence of supraventricular arrhythmias was 0.95%/year (95% CI = 0.42%/year to 1.59%/year). Two patients had ventricular arrhythmias, while one had atrioventricular blocks.

Out of the two patients who developed HF, one had a homogeneous pattern of myocardial iron overload at the baseline MRI (all segments with T2* < 20 ms), while the other one showed heterogeneous myocardial iron overload (some segments with T2* ≥ 20 ms and other segments with T2* < 20 ms) and no significant global heart iron (global heart T2* ≥ 20 ms).

The baseline RV mass index was > 52 g/m^2^ in all three patients who developed PH.

Table 1 presents the comparison of baseline characteristics and MRI parameters between patients who remained free of cardiovascular events and patients who experienced a cardiovascular event during the study. No significant differences were detected in the frequency of females, age, frequency of chelation, hematochemical parameters, number of transfusional units in the 12 months before the MRI scan, or cardiac and hepatic iron levels. All patients with cardiovascular complications were splenectomized, but the frequency of splenectomy was not different between the two groups. All LV functional parameters were comparable between the two groups, while patients who developed a cardiovascular complication exhibited significantly higher RV end-diastolic volume index (EDVI) and mass index. Compared to patients free of complications, patients with cardiovascular complications had a significantly higher frequency of LV replacement fibrosis, but no differences were detected in terms of the number of involved segments (3.33 ± 1.86 vs. 2.65 ± 1.53; *p* = 0.333) or the extent of myocardial fibrosis (3.55 ± 3.07% vs. 2.11 ± 1.76%; *p* = 0.273).

### 3.3. Prediction of Cardiovascular Events

Table 2 shows the results of the univariate Cox regression analysis. RV EDVI, RV mass index, replacement myocardial fibrosis, and right atrial (RA) area index were significant univariate prognosticators of cardiovascular complications.

Due to the low number of events, it was not possible to perform a multivariable analysis simultaneously including all univariable predictors. As the first step, a multivariable analysis, including the two RV size parameters (mass and volume), was carried out, and only the RV mass index was proven to independently predict cardiovascular diseases.

Then, a multivariable analysis, including RV mass index, RA atrial area, and replacement myocardial fibrosis, was conducted. In this model, RV mass index and LV replacement fibrosis emerged as independent predictors of cardiovascular outcomes (RV mass index: HR = 1.18, 95% CI = 1.08–1.29, *p* < 0.0001 and LV replacement fibrosis: HR = 6.26, 95% CI = 1.28–30.69, *p* = 0.024).

### 3.4. Best Cutoff of RV Mass Index for Risk Prediction

The RV mass index was not associated with age (R = −0.059; *p* = 0.570) but was significantly higher in males than in females (24.01 ± 6.23 g/m^2^ vs. 20.43 ± 6.36 g/m^2^; *p* < 0.0001).

The ROC curve analysis for identifying the best cutoff of RV mass index for risk prediction was performed separately in male and female patients.

**Figure 1:**
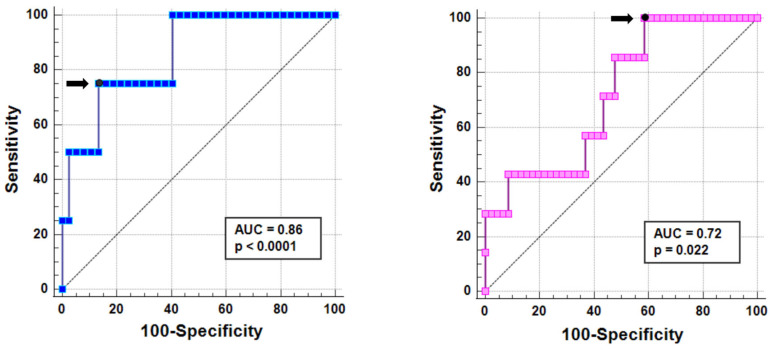
ROC curve analysis of RV mass index to predict cardiovascular events in males (**left**) and females (**right**). The black arrow indicates the best cut-off based on Yuden J’s statistics.

In males, an RV mass index > 27.2 g/m^2^ predicted the presence of future cardiovascular complications with a sensitivity of 75.0% and a specificity of 86.1% (*p* < 0.0001). The area under the curve was 0.86 (95% CI = 0.71–0.95) (Figure 1, left).

In females, an RV mass index > 18.2 g/m^2^ predicted the presence of future cardiovascular complications with a sensitivity of 100.0% and a specificity of 41.3% (*p* = 0.022). The area under the curve was 0.75 (95% CI = 0.61–0.86) (Figure 1, right).

### 3.5. Effect of Prior History of Cardiac Complications on Outcomes

No patient showing a cardiac complication at the time of the baseline MRI was included in the study, but 16 (8.9%) patients had a prior and resolved history of cardiac diseases. Specifically, six patients had a prior and resolved history of HF, nine of arrhythmias, and one of PH.

Patients who developed cardiovascular complications during the FU more frequently had a positive history of past complications than patients who did not develop cardiovascular diseases (38.9% vs. 5.6%; *p* = 0.002). The HR for previous cardiovascular complications predicting future cardiovascular complications was 12.78 (95% CI = 4.59–35.50, *p* < 0.0001).

When the previous arrhythmias were included in the multivariate model, the independent predictive factors of cardiovascular complications were RV mass index (HR = 1.13, 95% CI = 1.03–1.24, *p* = 0.012), LV replacement fibrosis (HR = 7.32, 95% CI = 1.36−39.51, *p* = 0.021), and previous cardiovascular complications (HR = 15.56, 95% CI = 2.29–105.87, *p* = 0.005).

### 3.6. Kaplan–Meier Survival Curves

To identify RV hypertrophy, the RV mass index was dichotomized according to the sex-specific cutoff values found in the ROC curve analysis.

**Figure 2:**
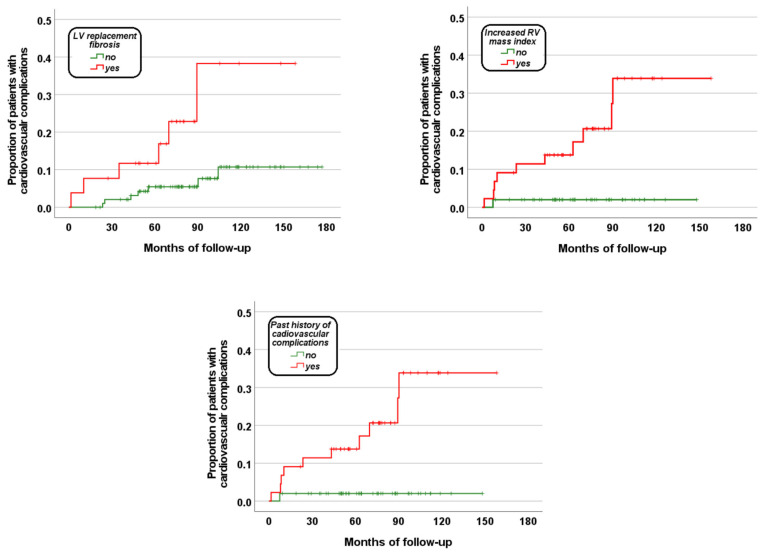
Kaplan–Meier curves showing the impact of each independent predictive factor on the development of globally considered cardiac complications.

Figure 2 shows the Kaplan–Meier survival curves describing the impact of each independent predictor on the development of cardiac complications. The log-rank test revealed significant differences in the curves for each predictor (RV hypertrophy: *p* = 0.004, LV replacement fibrosis: *p* = 0.006, and previous history of cardiovascular complications: *p* < 0.0001).

## 4. Discussion

To the best of our knowledge, this is the first study to evaluate the prognostic impact of multiparametric CMR, including RV mass assessment, in neo β-TDT patients.

The incidence of cardiac complications (HF + arrhythmias + PH) found in our neo-TDT patients (1.56%/year) was similar to that detected among the β-TDT patients (1.6%/year) treated since early childhood and enrolled in the MIOT Network in a comparable timeframe [41]. However, in neo-TDT, the incidence of HF was significantly lower (0.2%/year vs. 0.8%/year; *p* = 0.028), while no difference was detected in the incidence of supraventricular arrhythmias (0.9%/year vs. 0.7%/year; *p* = 0.568), which emerged as the most frequent cardiac complications of neo-TDT patients. The reduced incidence of HF in neo-TDT compared to TDT patients may be explained by the significantly lower cardiac iron burden (heart T2*: 34.85 ± 9.89 ms vs. 27.37 ± 12.44 ms; *p* < 0.0001). Cardiac siderosis plays a key role in the development of left ventricular dysfunction and symptomatic heart failure, and cardiac T2* has been demonstrated to be a powerful independent predictor of both non-fatal and fatal heart failure in TDT [41,42,43]. On the other hand, myocardial iron accumulation has a less pronounced impact on the development of supraventricular arrhythmias as compared to its role in the onset of cardiac failure [41,42]. The atrial myocardium might be more sensitive to iron deposition than the ventricle in the causation of arrhythmias. Unfortunately, T2* measurements of the thin atria are not robust.

As in optimally treated TDT populations [44,45], PH was not a major cardiac complication among neo-TDT patients. Appropriate transfusion therapy can effectively restore tissue oxygen supply and inhibit the production of dysfunctional native red blood cells, addressing key factors associated with PH such as hemolysis, hypercoagulability, tissue oxygen deficiency, and excess fluid volume [10,46,47].

In our cohort of neo-TDT patients, increased RV mass index and LV replacement fibrosis emerged as independent predictors of cardiovascular complications.

The RV has often been referred to as the “forgotten chamber” in the context of cardiac physiology and pathology, as its significance has historically been underestimated or overlooked. However, more recently, the prognostic and clinical significance of the RV in cardiovascular diseases has been largely recognized [48]. In addition to reflecting subtle LV abnormalities and pulmonary pathology [49,50], RV changes may directly contribute to cardiovascular risk. The Multi-Ethnic Study of Atherosclerosis (MESA) demonstrated in large cohorts of subjects without clinical cardiovascular disease at baseline that RV hypertrophy independently predicted an increased risk of clinically diagnosed HF [51] and atrial fibrillation [52]. The RV hypertrophy might function as a sensitive “barometer” of LV function, better reflecting increased LV end-diastolic pressure over time compared to a single quantitative CMR measurement of LV parameters like LV mass or EF [51]. Moreover, the RV’s morphology has been demonstrated to be associated with known processes implicated in the pathogenesis of atrial fibrillation, like systemic inflammation and neurohormonal activation, which influence myocardial fibrosis, atrial stretch, and modulation of ionic channel function [52,53]. Importantly, an increased RV mass index also demonstrated a significant prognostic value in patients affected by sickle-cell disease [54]. Although our results need to be validated and confirmed in further studies, they warrant including the RV mass assessment into the routine CMR of patients with hemoglobinopathies.

The prognostic significance of LV myocardial fibrosis by LGE has been established in ischemic and non-ischemic cardiomyopathies [55,56,57,58,59], and also in TDT [41], but this is the first study to demonstrate that myocardial fibrosis is an important prognostic marker of cardiovascular events in neo-TDT patients. Replacement fibrosis appears to be an irreversible process that represents a final common pathway across a breadth of pathologies [59]. Over time, the changes associated with myocardial fibrosis, including worsening ventricular systolic function, abnormal cardiac remodeling, and increased ventricular stiffness, may lead to LV failure and HF symptoms [60]. Moreover, the reduced ventricular compliance, resulting in compromised relaxation and elevated LV filling pressures, can cause an increase in left atrial pressures and structural remodeling, acting as a substrate for the onset of atrial fibrillation [61,62].

Unlike in TDT patients [41,42], we failed to detect a prospective link between myocardial iron overload and cardiac complications. This lack of association is likely due to the limited occurrence of significant myocardial iron overload and heart failure.

### Study Limitations

This study has some limitations.

Our results might have been underpowered due to the relatively small sample size and low incidence of cardiovascular outcomes. In particular, we could not consider the different types of cardiovascular events separately. Larger-scale studies may allow us to address this issue.

Information on iron burden, usually estimated through the transfusional iron intake, was not available.

T1, T2, and extracellular volume mapping techniques were not employed, since they were not available at the time of the patients’ enrollment. The inclusion of T1 mapping analysis could have been beneficial for enhancing the sensitivity in detecting mild or early myocardial iron overload [63,64].

We did not acquire three-dimensional high-resolution LGE sequences, which would have allowed us to accurately detect and quantify left atrial fibrosis, a common pathophysiological factor in the onset and maintenance of atrial fibrillation [65,66].

In the absence of symptoms or specific recommendations by a cardiologist, standard ECG was performed annually, and 24-hour ECG monitoring was performed every two years. Therefore, it is possible that asymptomatic episodes of atrial arrhythmias could have been missed.

## 5. Conclusions

Increased right ventricular mass index and replacement myocardial fibrosis emerged as strong independent predictors of cardiovascular complications in neo β-TDT patients. Therefore, implementing a comprehensive MR program that leverages its multiparametric capabilities has the potential to improve the prognosis of thalassemia patients through the early detection and management of those individuals at increased risk of cardiac complications.

## Figures and Tables

**Table 1:** Baseline demographic, clinical, and MRI findings in all neo-TDT patients and patients stratified according to the development of a cardiovascular event during follow-up.

**Variable**	**All Patients** **(N = 180)**	**No Cardiovascular Events** **(N = 162)**	**Cardiovascular Events** **(N = 18)**	***p*-Value**
Females, N (%)	106 (58.9)	96 (59.3)	10 (55.6)	0.762
Age (years)	38.25 ± 11.24	37.97 ± 11.59	40.78 ± 6.99	0.191
Splenectomy, N (%)	153 (85.0)	135 (83.3)	18 (100.0)	0.079
Chelation therapy, N (%)	170 (94.4)	153 (94.4)	17 (94.4)	1.000
Transfusional units in the last 12 months (N)	26.76 ± 10.19	26.69 ± 10.24	27.42 ± 10.26	0.801
Pre-transfusion hemoglobin (g/dL)	9.22 ± 0.79	9.22 ± 0.81	9.22 ± 0.63	0.847
Serum ferritin (ng/L)	1146.29 ± 987.58	1150.01 ± 999.39	1113.94 ± 905.19	0.906
MRI LIC (mg/g/dw)	9.77 ± 11.96	9.73 ± 12.33	10.09 ± 8.08	0.457
MRI LIC ≥ 3 mg/g/dw, N (%)	132 (73.3)	118 (72.8)	14 (77.8)	0.784
Global heart T2* (ms)	34.85 ± 9.89	35.30 ± 9.64	30.74 ± 11.46	0.052
Global heart T2* < 20 ms, N (%)	21 (11.7)	17 (10.5)	4 (22.2)	0.235
No. of segments with T2* < 20 ms	2.16 ± 4.72	2.02 ± 4.55	3.39 ± 6.12	0.556
LV EDVI (mL/m^2^)	90.23 ± 18.62	89.38 ± 18.18	97.59 ± 21.25	0.076
LV mass index (g/m^2^)	60.84 ± 13.38	60.60 ± 13.51	62.88 ± 12.33	0.486
LV EF (%)	63.55 ± 6.39	63.51 ± 6.29	63.89 ± 7.40	0.811
RV EDVI (mL/m^2^)	84.98 ± 19.87	83.35 ± 18.82	99.11 ± 23.49	0.013
RV mass index (g/m^2^)	21.99 ± 6.52	20.99 ± 4.38	29.57 ± 12.91	0.014
RV EF (%)	64.27 ± 6.61	64.54 ± 6.48	61.94 ± 7.47	0.116
LV replacement fibrosis, N (%)	26/125 (20.8)	20/112 (17.9)	6/13 (46.2)	0.017
LA area index (cm^2^/m^2^)	13.89 ± 2.58	13.77 ± 2.60	14.71 ± 2.28	0.105
RA area index (cm^2^/m^2^)	12.74 ± 2.22	12.61 ± 2.08	13.70 ± 2.89	0.132

N = number, MRI = magnetic resonance imaging, LIC = liver iron concentration, LV = left ventricular, EDVI = end-diastolic volume index, EF = ejection fraction, RV = right ventricular, LA = left atrial, RA = right atrial.

**Table 2:** Results of univariate and Cox regression analysis for predictors of cardiovascular complications.

	**Univariate Analysis**	
	**HR (95% CI)**	***p*-Value**
Female gender	0.83 (0.33–2.11)	0.694
Age	1.03 (0.99–1.08)	0.145
Splenectomy	25.34 (0.08–8587.24)	0.277
Chelation therapy	0.90 (0.12–6.79)	0.920
Pre-transfusion hemoglobin	0.98 (0.53–1.83)	0.952
Serum ferritin	1.00 (0.99–1.00)	0.725
MRI LIC	0.99 (0.97–1.04)	0.963
Global heart T2*	0.97 (0.93–1.01)	0.086
No. of segments with T2* < 20 ms	1.05 (0.97–1.13)	0.250
LV EDVI	1.02 (0.99–1.05)	0.116
LV mass index	1.01 (0.98–1.05)	0.504
LV EF	1.03 (0.95–1.11)	0.469
RV EDVI	1.04 (1.02–1.06)	0.001
RV mass index	1.14 (1.08–1.20)	<0.0001
RV EF	0.96 (0.89–1.03)	0.209
LV replacement fibrosis	4.21 (1.39–12.69)	0.011
LA area index	1.17 (0.98–1.39)	0.081
RA area index	1.23 (1.01–1.51)	0.044

HR = hazard ratio, CI = confidence interval, MRI = magnetic resonance imaging, LIC = liver iron concentration, LV = left ventricular, EDVI = end-diastolic volume index, EF = ejection fraction, RV = right ventricular, LA = left atrial, RA = right atrial.

## Data Availability

The data underlying this article cannot be shared publicly due to privacy reasons. The data will be shared upon reasonable request to the corresponding author.
